# Cancer mortality in IBM Endicott plant workers, 1969–2001: an update on a NY production plant

**DOI:** 10.1186/1476-069X-7-13

**Published:** 2008-04-28

**Authors:** Richard W Clapp, Kate Hoffman

**Affiliations:** 1 Department of Environmental Health, Boston University School of Public Health, 715 Albany Street, Boston, MA 02118, USA

## Abstract

**Background:**

In response to concerns expressed by workers at a public meeting, we analyzed the mortality experience of workers who were employed at the IBM plant in Endicott, New York and died between 1969–2001. An epidemiologic feasibility assessment indicated potential worker exposure to several known and suspected carcinogens at this plant.

**Methods:**

We used the mortality and work history files produced under a court order and used in a previous mortality analysis. Using publicly available data for the state of New York as a standard of comparison, we conducted proportional cancer mortality (PCMR) analysis.

**Results:**

The results showed significantly increased mortality due to melanoma (PCMR = 367; 95% CI: 119, 856) and lymphoma (PCMR = 220; 95% CI: 101, 419) in males and modestly increased mortality due to kidney cancer (PCMR = 165; 95% CI: 45, 421) and brain cancer (PCMR = 190; 95% CI: 52, 485) in males and breast cancer (PCMR = 126; 95% CI: 34, 321) in females.

**Conclusion:**

These results are similar to results from a previous IBM mortality study and support the need for a full cohort mortality analysis such as the one being planned by the National Institute for Occupational Safety and Health.

## Background

There have been a number of recent studies of cancer incidence [1] and mortality [2] and other health outcomes in workers in the semiconductor and computer manufacturing industries. Some of these have focused on workers in a particular plant or group of similar plants and one [3] examined the mortality experience of employees at multiple plants for a large company. The exposures vary at these manufacturing and fabrication facilities, and they have changed over time as the technology and work processes have developed or become automated [4]. The meaning and significance of these studies have been discussed in review articles and commentaries [5-7].

Another concern that has arisen is pollution in a community near an IBM manufacturing plant in Endicott, NY. This has resulted in an evaluation of trichloroethylene in groundwater and vapor intrusion into the basements of nearby homes and businesses. Attention to this source of exposure has led employees of this IBM plant to request a study of cancer and other health effects in former workers. Although this plant was one of the original IBM manufacturing plants and a variety of products were made there over the past several decades, the most recent activities were the production of printed circuit boards. It therefore had some chemical and physical exposures that were similar to other manufacturing facilities, including the IBM San Jose plant.

In response to concerns expressed by employees, we conducted a supplementary analysis of data from a previous publication on mortality among the U.S. employees of the IBM company [3]. One of us (RWC) attended a meeting in the community near the plant in Endicott, NY and heard the concerns from former IBM employees in the audience about cancer in workers, in particular. This former IBM facility had produced a variety of products since it began operations in 1911, including calculating machines, typewriters, guns, printers, automated machines for banks, printed circuit boards and chip packaging. The purpose of the supplementary analysis of mortality data for this plant was to see if there were patterns of cancer mortality similar to those seen in other IBM manufacturing workers during the years 1969–2001. IBM sold the Endicott microelectronics facility in 2002.

## Methods

The present supplementary analysis was carried out in response to workers' desires for information. It is based on the IBM Corporate Mortality File (CMF) which was produced after a court order during litigation and used in a previous analysis, but which did not look at the Endicott plant workers separately. The CMF contained records for deceased individuals who had been employed in an IBM facility for five years or more, who were actively employed or receiving retirement or disability benefits at time of death, and whose families had filed a claim for death benefits in their Corporate Mortality File (CMF). Within the CMF, underlying causes of death were coded to the Ninth Revision of the International Classification of Disease (ICD 9). As in the previous study, data from the CMF were joined to work history data from the IBM Corporate Employee Resource Information System (CERIS) that included a location code for place of employment. Death records of individuals who had worked at Endicott between 1969 and 2001 were selected using seven location codes that were associated with the facility over time. Proportional cancer mortality (PCMR) analysis [8] was conducted using publicly available data for the state of New York as a standard of comparison [9]. Unfortunately, state cancer mortality data could not be located for the entire study period; however, comparison data for the years 1979 to 1998 were used in the PCMR analyses. Proportional cancer mortality ratios were calculated from the ratio of the observed number of deaths due to a particular cancer type among the workers to the expected number of deaths of that type in each sex. For each PCMR, 95% Poisson confidence intervals were also calculated.

## Results

From the CMF and CERIS files, we identified 360 Endicott employee deaths. Of these, 115 deaths were attributable to cancer. The number of cancer deaths by age group and sex is presented in Table 1; the number of deaths by cancer type and sex is presented in Table 2.

**Table 1:** Endicott cancer deaths by age and sex.

**Age Group**	**Females**	**Males**
**35–44 years**	1	1
**45–54 years**	1	15
**55–64 years**	6	28
**65–74 years**	6	55
**75–84 years**	1	1

**Table 2:** Endicott cancer deaths by type and sex.

	**Females**	**Males**
**All Cancers**	15	100
**Bladder**	0	1
**Brain**	0	4
**Breast**	4	1
**Colon**	0	7
**Endocrine Glands**	0	1
**Esophagus**	1	4
**Gallbladder**	1	2
**Kidney**	0	4
**Larynx**	1	0
**Liver**	0	1
**Lung**	3	35
**Lymphoma**	0	9
**Melanoma**	0	5
**Multiple Myeloma**	0	3
**Nasal cavities, middle ear, and accessory sinuses**	0	1
**Nasopharynx**	1	0
**Oropharynx**	0	1
**Ovary**	1	0
**Pancreas**	1	4
**Rectum**	1	3
**Stomach**	0	4
**Without Specific Site**	1	10

The PCMR results for those cancer types with at least four deaths in one sex are presented in Table 3. The full analysis is provided as an appendix [see Additional file 1]. Mortality due to melanoma (PCMR = 367; 95% CI: 119, 856) and lymphoma (PCMR = 220; 95% CI: 101, 419) were significantly increased in males. Modestly increased mortality due to kidney cancer (PCMR = 165; 95% CI: 45, 421) and brain cancer (PCMR = 190; 95% CI: 52, 485) in males were also observed. In females, a modest increase in breast cancer (PCMR = 126; 95% CI: 34, 321) was observed.

**Table 3:** Endicott cancer PCMRs by type and sex.

	**Males**					**Females**				
	**Observed**	**Expected**	**PCMR**	**Lower C.I.**	**Upper C.I.**	**Observed**	**Expected**	**PCMR**	**Lower C.I.**	**Upper C.I.**

**All Cancers**	100	100.0	-	-	-	15	15.0	-	-	-
**Esophagus**	4	3.6	111.6	30.3	285.6	1	0.2	524.0	13.3	2918
**Stomach**	4	3.9	102.8	33.3	239.5	0	-	-	-	-
**Colon**	7	9.6	72.6	29.1	149.6	0	-	-	-	-
**Pancreas**	4	5.3	76.0	20.7	194.6	1	0.8	132.7	3.4	739.1
**Lung**	35	34.2	102.5	71.4	142.4	3	3.2	94.7	19.5	276.6
**Melanoma**	5	1.4	367.3	119.0	855.7	0	-	-	-	-
**Breast**	1	0.1	728.0	18.4	4054	4	3.2	125.5	34.1	321.3
**Kidney**	4	2.4	164.5	44.7	421.1	0	-	-	-	-
**Brain**	4	2.1	189.5	51.6	485.2	0	-	-	-	-
**All Lymphoma**	9	4.1	220.4	101.0	418.8	0	-	-	-	-

## Discussion

Our results, although based on a small number of deaths, suggest an increased risk of several types of cancer among employees of the Endicott facility. According to a NIOSH feasibility study [10] completed over a year ago, there were 28,000 workers employed at the Endicott plant for at least a year after 1965. A large percentage (44%) were potentially exposed to one or more carcinogens, including asbestos, benzene, cadmium, nickel compounds, vinyl chloride or probable carcinogens such as tetrachloroethylene, trichloroethylene, PCBs, ortho-toluidine and ultraviolet light. A variety of other solvents were used, and chlorinated solvents were eventually phased out beginning in the 1980s. NIOSH has concluded that a cohort study of the Endicott employees is feasible and is currently developing a protocol to conduct the study.

Other studies of workers in the semiconductor and computer manufacturing industry are underway in the U.S. and Europe. The literature to date is quite sparse, and it is anticipated that these new studies will shed additional light on risks of cancer and other diseases in this important international industry. The challenge will be to evaluate exposures as they change over time and incorporate this information into epidemiologic analyses of subsequent health risks. Safer materials and production methods appear to be needed irrespective of the ultimate outcome of the studies.

## Conclusion

This mortality analysis updates previous results presented for all IBM employees and for sub-groups of manufacturing workers and workers at other specific plants. In the present analysis, several types of cancer were elevated as a cause of death in Endicott workers, compared to deaths in New York State during the same time period. The PCMR results in Table 3 show significantly elevated mortality due to melanoma and lymphoma, and elevated but not significant mortality due to breast cancer, kidney cancer and brain cancer. These results are similar to the results seen in IBM manufacturing plants such as the San Jose, CA plant in previous analyses.

These results are based on small numbers of deaths, and they do not include deaths in workers who were not eligible to be in the Corporate Mortality File. The similarity in the pattern of mortality and the similarity in some of the chemical exposures in the Endicott plant suggest that there may be more information to be learned from a full cohort study such as the one being proposed by NIOSH.

## Abbreviations

CERIS: IBM Corporate Employee Resource Information System; PCMR: Proportional cancer mortality ratio; CI: Confidence Interval; IBM: International Business Machines; CMF: Corporate Mortality File; ICD-9: International Classification of Diseases, 9^th ^revision.

## Competing interests

The first author (RWC) was previously paid a consultancy by the plaintiffs' law firm; the law firm did not design or conduct the study, nor review or approve the manuscript. The author received no remuneration for the preparation of this manuscript.

## Authors' contributions

RWC was responsible for the overall conception and presentation of the work. KH calculated the PCMRs and wrote the methods description. Both authors read and approved the final manuscript.

## Supplementary Material

Additional file 1 Observed and expected cancer deaths, by age group, sex and cause of death, Endicott workers, 1969–2001. The data provided are the numbers of deaths and calculated PCMRs and confidence intervals for all cancers observed in the Endicott workers. Click here for file
